# From Outbreaks to Artificial Intelligence: A Comprehensive Review of Monkeypox Virus Epidemiology, Diagnosis, Treatment, Vaccination, and Deep Learning Applications

**DOI:** 10.1155/jotm/6688914

**Published:** 2024-12-30

**Authors:** Shahed Ahmadi, Mahdi Amirzadeh, Mousa Ahmadi, Saeed Soleiman-Meigooni

**Affiliations:** ^1^Department of Clinical Pharmacy, School of Pharmacy, Shahid Beheshti University of Medical Sciences, Tehran, Iran; ^2^Department of Infectious Disease, Faculty of Medicine, Aja University of Medical Sciences, Tehran, Iran

**Keywords:** epidemiology, monkeypox virus, outbreak, treatment, vaccination

## Abstract

**Objectives:** After the global impact of the COVID-19 pandemic, concerns over virus transmission have risen. A state of health emergency was declared in 2022 due to Clade 2 of the monkeypox (MPOX) virus. In August 2024, another emergency was declared by the World Health Organization (WHO) because of the widespread Clade 1b, which caused a more severe and lethal disease. This review synthesizes current MPOX knowledge to assist policymakers, clinicians, and researchers in developing effective diagnostics, therapeutic interventions, vaccination strategies, and outbreak management.

**Methods:** This systematic review study searched for articles on virus epidemiology, virology, clinical features, transmission routes, available drugs and vaccines, and new artificial intelligence (AI) applications in diagnosis and drug discovery.

**Results:** MPOX virus is a 200–250-nm, double-stranded DNA (ds-DNA) virus that causes smallpox-like skin lesions. Tecovirimat is the primary drug for severe cases, especially in people with suppressed immune systems. Smallpox vaccines can help prevent MPOX infection because of the genetic similarities between the MPOX and smallpox viruses. AI-based models can assist medical teams in promptly diagnosing MPOX skin lesions, improving decision-making for treatment.

**Conclusion:** This review highlights the importance of using traditional public health knowledge alongside modern AI to manage MPOX outbreaks effectively. It shows that we need strong public health policies, focused interventions, and ongoing research on how AI can help control infectious diseases.

## 1. Introduction

Monkeypox (MPOX) is a severe and potentially life-threatening zoonotic infectious disease found in Central and West Africa, but it is usually self-limiting [1]. This tropical viral infectious disease is caused by the family *Poxviridae*, the subfamily *Chordopoxvirinae*, and the genus *Orthopoxvirus*. These are among the largest known DNA viruses, in both size and genetic complexity. Its endemic prevalence in tropical regions, such as Africa, and the characteristics of the pathogen that causes it highlight the tropical and infectious nature of this disease. It has two distinct strains: Clade 1, the Central African strain, which causes a more severe disease with a higher fatality rate, and Clade 2, or the West African strain, which tends to result in a milder illness. After an incubation period of 1–3 weeks, nonspecific symptoms such as fever, headache, lymphadenopathy (more typical in MPOX infection), and muscle pain appear, followed by rashes. In the 2022 outbreak, skin lesions were mainly reported in the anogenital region [2]. It spreads through contact with infected animals, bodily fluids, or contaminated objects. Pregnant women, children, those with an eczema history, and immunocompromised individuals are at higher risk [3, 4]. MPOX is diagnosed through polymerase chain reaction (PCR) testing of skin or mucous-membrane samples [5].

Treatment for MPOX mainly involves supportive care. Antiviral drugs such as tecovirimat, cidofovir (CDV), and brincidofovir (BCV) are utilized under specific conditions [6]. Developing new drug delivery systems, such as tecovirimat adsorbed onto carbon microtubes, shows promise for more effective treatment [7]. Preventive measures are crucial, including avoiding direct contact with infected persons and adhering to strict hygiene and quarantine protocols. Smallpox vaccines provide cross-protection against MPOX due to genetic similarities, while mRNA-based vaccines are being developed for targeted prevention [8].

From January to August 2024, over 15,600 MPOX cases and 537 deaths were reported globally, representing a significant public health challenge. The World Health Organization (WHO) attributed this outbreak to the highly virulent Clade 1b strain, associated with severe clinical manifestations and elevated mortality rates. The outbreak primarily affected East, Central, West, and Southern Africa, with high cases reported in Burundi, Kenya, Rwanda, Uganda, the Central African Republic (CAR), Ivory Coast, and South Africa. The rapid spread within these regions highlighted the need for coordinated containment strategies and improved healthcare infrastructure to manage such outbreaks.

In August 2024, Sweden reported the first case of Clade 1b outside Africa. The case involved an individual who had recently traveled to an affected African region, underscoring the significant role of international travel in the global spread of infectious diseases. This development emphasized the need for policymakers to strengthen border health protocols, implement targeted surveillance measures, and enhance global collaboration to prevent cross-border transmission. The 2024 outbreak has reinforced the importance of integrating travel-related health policies with outbreak management strategies to mitigate the risks of highly transmissible pathogens.

This review brings together the latest knowledge about MPOX, covering key areas such as its epidemiology, clinical features, diagnostic methods, treatment options, vaccination strategies, and the emerging role of artificial intelligence (AI) in disease management. By combining traditional public health approaches with cutting-edge AI innovations, this review provides valuable insights for policymakers, clinicians, and researchers. It aims to support decision-making, identify research gaps, and enhance preparedness for managing MPOX and similar infectious diseases. This comprehensive perspective contributes to global efforts to control outbreaks and strengthen public health responses.

## 2. Method and Material

Between March and August 2024, we conducted a comprehensive literature search employing advanced search techniques on PubMed, Scopus, and Google Scholar to identify appropriate studies on MPOX. Our priority was on articles published from 2022 onward, while earlier publications were included selectively to provide historical context and foundational information. The search strategy utilized combinations of keywords such as “monkeypox epidemiology,” “clinical features of monkeypox,” “PCR monkeypox,” “monkeypox treatment options,” “monkeypox vaccines,” and “artificial intelligence in infectious diseases.” Boolean operators (AND, OR) were employed to refine results, for instance, “monkeypox AND (vaccine OR vaccination)” and “AI AND monkeypox AND diagnosis.”

We defined strict inclusion and exclusion criteria to ensure the quality and relevance of the studies. Peer-reviewed articles, case reports, case series, and studies involving human subjects were included, particularly those addressing epidemiology, clinical manifestations, diagnosis, treatment, vaccination, and AI applications. Non–peer-reviewed articles, studies focusing exclusively on animal models, articles with insufficient data, and meta-analyses were excluded.

We adopted a double-masked screening process to minimize selection bias. Two independent reviewers assessed article titles and abstracts. Discrepancies were resolved through discussion or consultation with a third reviewer. Full-text articles were evaluated using a predefined checklist to ensure methodological rigor and relevance. To further reduce bias, a fourth team member reviewed the final selection and data extraction for accuracy. Utilizing multiple databases also ensured the comprehensive coverage of the available literature.

**Figure 1:**
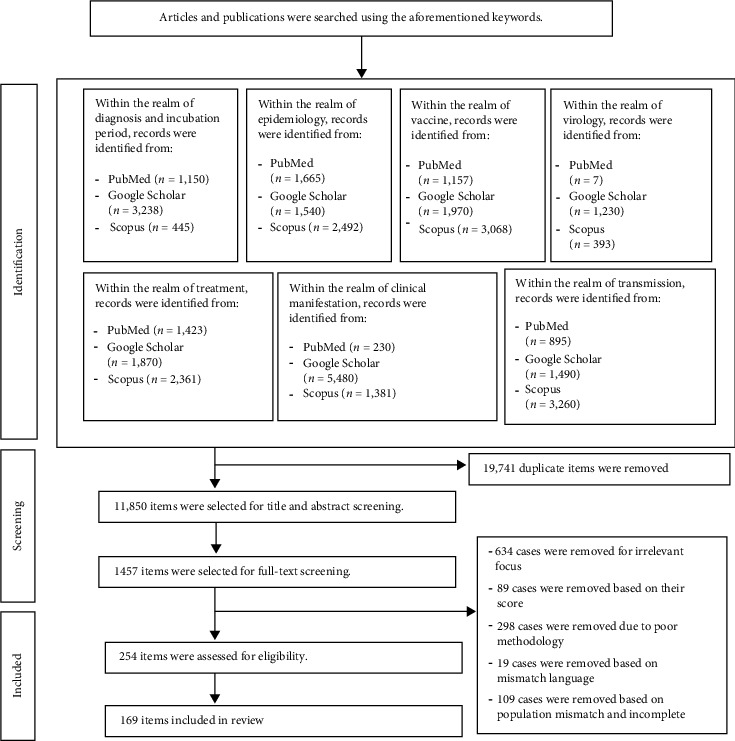
Flowchart of the study selection process.

Data extraction followed a systematic approach, capturing key details such as study design, geographical location, sample characteristics, clinical findings, and significant outcomes. A standardized extraction form was used to maintain uniformity across studies. The findings were synthesized qualitatively to provide a thorough overview of MPOX's epidemiology, clinical features, diagnostic methods, treatment strategies, and AI applications. The flowchart (Figure 1) clearly outlines the thorough and methodical process we use for selecting articles, ensuring quality and relevance.

## 3. Results

### 3.1. History and Epidemiology

In 1958, the virus was named MPOX after a new orthopoxvirus outbreak among cynomolgus monkeys in Copenhagen [9]. The first case of human infection with the MPOX in the Democratic Republic of the Congo (DRC) was observed in 1970 in a 9-month-old boy [10]. In the 1970s, MPOX outbreaks in the DRC spread through human-to-human transmission and close contact with infected animals during the hunts in warm seasons and hunting areas [10, 11]. The increase in human-to-human transmission raises apprehensions about the potential for widespread zoonotic disease. As a result, between 1981 and 1986, the WHO reported 338 suspected cases of the disease and 33 deaths through the implementation of surveillance programs [12]. Subsequently, positive cases decreased dramatically over the following six years, with only 13 cases registered in Cameroon, Gabon, and the DRC from 1986 to 1992 [12, 13]. Kasaï-Oriental Province in the DRC was the site of the next major outbreak two years following 1992, with 511 suspected cases in the Katako-Kumba region and 24 in the Lodja health zone [13–16]. Between 1998 and 2001, the DRC and CAR experienced an ongoing MPOX outbreak, during which the Ministry of Health of DRC documented 1265 cases over the three years [17, 18]. In 2003, the United States reported the first human cases of MPOX outside Africa, with 72 instances of Clade 2 MPOX, with a median age of 28 years traced back to prairie dogs that had been infected by rodents transported from Ghana to Texas [19]. From September to December 2005, cases spread in Sudan's Unity State, with 760 cases reported across nine locations in Congo by 2007 [12]. No outbreaks occurred outside Africa until 2021. In Africa, outbreaks were reported in the DRC, Sierra Leone, and CAR between 2010 and 2016. These included a small outbreak in the Likouala region of the DRC, an outbreak in Bo City, Sierra Leone, and reported cases in Bangassou, in CAR. A more widespread outbreak occurred in the DRC between 2014 and 2016, with 587 suspected cases detected during the country's surveillance program [18]. The largest outbreak of MPOX in West Africa occurred between 2017 and 2018, with 244 cases following the infection of an 11-year-old child in Nigeria [12]. The travel of suspected cases of Clade 2 from Nigeria to the United Kingdom and Israel caused the 2018 outbreak in these two countries [20, 21]. In July 2021, a middle-aged American man with a history of traveling to Nigeria was reported as an infection-positive case in the United States [19]. As vaccination against smallpox declined, global immunity to orthopoxviruses also decreased. In March 2022, while the world was still grappling with COVID-19, six confirmed cases of MPOX and two deaths were reported in the CAR. From the beginning of 2022 until April 17, the DRC registered 1152 suspected cases with 55 deaths [19].

In 2022, a major global outbreak of MPOX occurred as the infection spread from Africa to Europe and America. The majority of cases were among men who have sex with men (MSM) [22], many of whom had not traveled to areas where the disease is endemic. It is vital to clarify the epidemiological links between instances in different regions and the role of asymptomatic in spreading the virus [12].

Comparatively to 2022, the practically steady and declining trend of obtaining this virus in Africa in 2023 is seen. Between the start of 2024 and the writing of this article in August, there have been more than 15,600 positive cases with 537 deaths. Unlike the 2022 outbreak with Clade 2, the WHO has linked this new outbreak in the DRC to the more lethal strain of the virus, Clade 1b, to neighboring nations [23]. The new outbreak began in July of this year and is currently affecting East, Central, West, and South Africa. The nations that reported positive cases of the new disease in the most recent outbreak were Burundi, Kenya, Rwanda, and Uganda in East Africa; the CAR in Central Africa; Ivory Coast in West Africa; and South Africa [24]. On August 14, 2024, the WHO again issued a public health emergency due to the general spread of the most current strain [23]. The clade has quickly spread to countries outside Africa, marking a significant global impact. On August 15, 2024, Sweden became the first nation outside of Africa to record a positive case of the hazardous clade of MPOX in someone who had recently resided in Africa [25]. Pakistan [26]and the Philippines [27] also announced new positive cases of the Clade 1b following Sweden's case.

### 3.2. Virology

The MPOX virus is a double-stranded DNA (ds-DNA) virus belonging to the *Poxviridae* family and genus *Orthopoxviruses*. Its vast persistence in the zoonotic cycle and diverse range of hosts set it apart from other members of this family (e.g., cowpox, variola virus, and smallpox) [28, 29]. It was known by this name since 1958 after infecting cynomolgus monkeys [9]. There are now two different clades of the MPOX virus. Clade 1 is the leading cause of MPOX infections in Central Africa and the Congo Basin [30–32], with a case-fatality rate ranging from 1% to 12% [33]. Conversely, the most common strain in West Africa is Clade 2, which has a much lower case-fatality rate of about 0.1% [32, 34, 35]. The 2022 global outbreak of MPOX was similarly linked to Clade 2 [36], which exhibited distinct severity and clinical manifestations when compared to Clade 1. The differences in case-fatality rates and infection severity between these two clades are caused by a slight genetic variance of around 0.5% in their genomic sequences [30]. Host–response modifier (HRM) genes, such as the complement pathway inhibitor enzyme gene (PICE or MOPICE), exhibit diversity among the clades of the MPOX virus, contributing to the differences between them [32, 37]. As suggested by animal studies, the absence of this gene in Clade 2 is responsible for the lower case-fatality rate observed in this clade [38]. The B.1 lineage, a subgroup of Clade 2b (known as Clade 3), was responsible for the 2022 global outbreak, and the only reported case of MPOX in Iran (Khuzestan) was infected with this lineage [12, 39]. The MPOX genome is a 197-kilobase pair ds-DNA linear genome with inverted terminal repeats (ITRs) at both ends. It encodes approximately 200 proteins essential for viral particle formation, DNA replication, and completing the viral structure within the host cell [33]. The MPOX virus has a brick-shaped structure measuring 200–250 nm. Its surface is covered in tubular filaments, and a biconcave core houses the viral genome, similar to those of variola and vaccinia viruses.

### 3.3. Diagnose

Diagnosing MPOX without clinical testing is especially important in low-resource settings where access to healthcare facilities and laboratory tests is limited. Recognizing key symptoms, such as fever, headache, muscle aches, back pain, swollen lymph nodes, and a distinctive rash that progresses through various stages, can help identify the disease early. This approach can reduce the financial burden on families and ensure timely care. Social and environmental factors play a major role in the spread of MPOX. In regions where the disease is common, such as parts of Africa, people often live in close contact with wildlife, increasing the risk of catching the virus. Activities like hunting and eating bushmeat expose individuals to infected animals, such as rodents and primates, which are the primary carriers of the virus. Communities living near forests or rural areas where these animals are common face a higher risk of infection. Poor living conditions, such as lack of clean water, overcrowded homes, and limited access to healthcare, make it easier for the disease to spread within families and neighborhoods. Migration, travel, and large gatherings also contribute to the virus moving into new areas. Raising awareness about these risks, encouraging preventive actions, and training healthcare workers to identify symptoms can make a big difference in controlling MPOX outbreaks and protecting vulnerable communities.

#### 3.3.1. Laboratory Diagnosis

As advised by the WHO, other potential diagnoses such as syphilis, Orf, mycosis, and drug sensitivity reactions should also be considered in the diagnosis of MPOX infection [40]. Real-time PCR (RT-PCR) is the most crucial test for virus detection, with laboratory guidelines recommending the targeting of at least two common genes among different strains of the disease. This adaptability is evident in the targeting of genes such as the TNF receptor gene, *E9L* DNA polymerase gene [41], *B6R* envelope protein gene [42], and DNA-dependent RNA polymerase gene [40], along with proposed target proteins including polymerase subunit 18, *B7R* [43], *FL3* [43], *N3R* [40], *C3L* complement-binding protein [40], *CP* protein gene [44], and *O2L* gene [40, 45]. Other less common identification methods include electron microscopy following staining to identify the virus in lesion samples [46] and detecting specific viral antigens through immunohistochemistrical methods [47].

Because the virus proliferates in mammalian cell lines, including HeLa, Vero, BSC-1, and RK-13, virus culture using these cell lines is one of the more established diagnostic methods [48]. Diagnosing MPOX with specific serological tests is challenging due to potential cross-reactivity with other orthopoxviruses. Assessing (acute immunoglobulin) IgM in the first week or (late immunoglobulin) IgG in paired samples 21 days apart can significantly impact patient care and aid in diagnosis [49].

### 3.4. Incubation Period

According to reports from the 2022 MPOX outbreak, the illness typically takes 5 to 13 days to incubate [50]. However, the length of time has varied depending on the region. The incubation period ranged from 6.5 to 10.9 days (with a 95% confidence interval [CI]; average 9.1 days) among patients tested in Italy in May and June of 2022 [51]. Compared to the 2005 outbreak in the United States, which lasted between 11 and 18 days, it seems that the incubation time for MPOX infections in 2022 has changed [52]. Furthermore, several factors affect the incubation period, like the infection route. For instance, compared to infection through sexual contact, the incubation time is shortened by being bitten or scratched [53]. Additionally, some reports indicate that this period can extend to 21 days [54].

### 3.5. Pathogenesis

**Figure 2:**
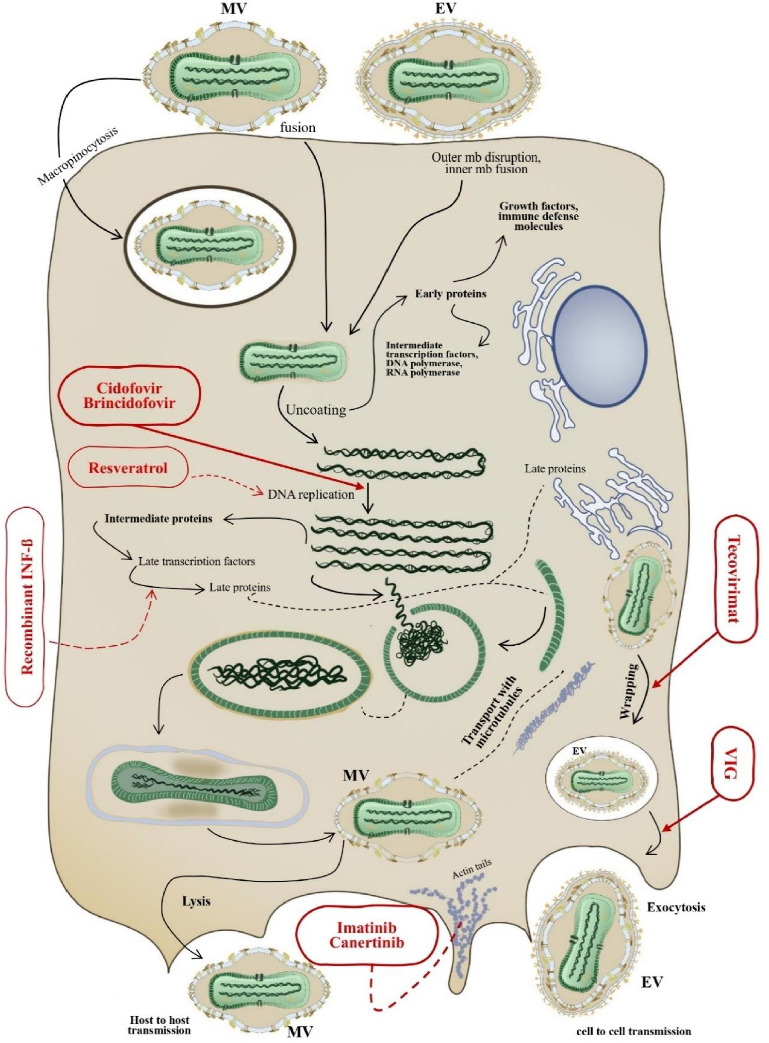
The reproduction cycle of the MPOX and the activation sites of different therapeutics against it; EVs, extracellular virions; MVs, mature virions; VIG, Vaccinia Immune Globulin.

The method of virus entry (via the respiratory system or the skin) and the specific clade of MPOX can affect the clinical manifestation of infection. The virus begins reproducing after it invades the host's respiratory system and mucous membranes in the throat and mouth through the respiratory route. Likewise, after exposure to MPOX in the dermis, keratinocytes, fibroblasts, and endothelial cells can undergo this process [55, 56]. Immunohistochemistry of skin lesion biopsies can reveal intraepidermal, intrafollicular, intravascular, interstitial, and perivascular neutrophils (MPO), T-cell infiltrates (CD3), cytotoxic T cells (CD4 and CD8), and macrophages (CD68) [47]. The pathophysiology of MPOX infection drafts two viremias. During primary viremia, the virus replicates at the site of infection and spreads to the lymph nodes, leading to submandibular, cervical, or inguinal lymphadenopathy. After secondary viremia, the virus disseminates to organs through the bloodstream [57]. The virus variant significantly affects disease manifestation. In Clade 1 of MPOX, the virus replicates in the respiratory epithelium during the incubation period before spreading to lymphoid organs such as the spleen and liver and then can be detected in the blood after the incubation period [58–60]. But primate models, infected by Clade 2, exhibited that the replication of virus befalls in just cutaneous and lymphoid system [33, 60]. This is why the clade of MPOX has higher pathogenicity. The replication cycle of the MPOX virus begins with the virus entering the host cell via micropinocytosis or direct fusion with the plasma membrane. Crucial for the attachment of the virion are key components such as exterior virion proteins, surface glycosaminoglycans, and extracellular matrix elements [61]. Upon entry, the virus damages its outer membrane, releasing its core into the cytoplasm for uncoating. Early gene expression generates essential early proteins, such as intermediate transcription factors, DNA polymerase, and RNA polymerase, required for viral replication and immune response control. The virus's DNA replicates within specialized areas of the cytoplasm, producing multiple copies of the viral genome. The virus synthesizes the intermediate proteins and late transcription factors to produce immature virions (IVs) and then mature to become mature virions (MVs). The Golgi apparatus transports some MVs, envelops them, and transforms them into wrapped virions (WVs). WV is carried to the cell's outside edge, either discharged as extracellular virions (EVs) or remained as cell-associated enveloped virions (CEVs) for cell transmission. Cell lysis can also release MVs, facilitating their transfer from one host to another. This process facilitates effective duplication and dissemination, resulting in observable clinical manifestations such as skin rash and swelling of the lymph nodes [62].  Figure 2 illustrates this process in detail, providing a more precise understanding.

### 3.6. Immune Response

The immune response to human MPOX infection involves both innate and adaptive immunity [63]. The innate immune system is triggered in response to viral infections. This activation occurs mainly through the release of proinflammatory cytokines such as tumor necrosis factor-alpha (TNF-*α*), interleukin-1 (IL-1), and interferons (INFs) by dendritic cells, natural killer cells, and macrophages. These cytokines serve to signal and attract more immune cells to the site of infection. This coordinated immune response aims to contain and eliminate the pathogen, ensuring a thorough and efficient defense [64]. Both CD4+ (helper T cells) and CD8+ cytotoxic T cells are activated by MHC Class I antigens from macrophages and dendritic cells. CD4 T cells help B cells become plasma cells that secrete antibodies. CD8+ T cells activated by interleukins, granzyme B, and perforin become more cytotoxic. This synchronized response kills virus-infected cells and provides a strong resistance against viral infections [65, 66]. Toll-like receptors (TLRs), NOD-like receptors (NLRs), C-type lectin receptors (CLRs), and RIG-1-like receptors (RLPs) are pattern recognition receptors (PRPs) that play a crucial role in the innate immune system. They are responsible for identifying a wide range of molecules associated with damaged cells [67]. Upon the activation of PRPs, a series of processes are initiated, resulting in the activation of transcription factors associated with inflammation, such as nuclear factor-kappa B (NF-*κ*B) and INF regulatory factors (IRFs) [68]. On the other hand, the virus has effective ways to dodge the immune system. A47R is a protein produced by MPOX that can interact with intracellular adaptors of TLRs and suppress the upregulation of NF-*κ*B expression, which leads to the inhibition of apoptosis regulating via interaction with the I*κ*B kinase (IKK) complex that is necessary for the activation of NF-*κ*B and has a critical role in control of programmed cell death [38, 68, 69]. MPOX employs soluble cytokines to evade the immune system. These cytokines competitively bind to the immune system's cytokines, hindering the expression of antiviral genes and facilitating viral replication. This is achieved by disrupting the activated downstream signaling pathway when cytokine receptors are occupied. Additionally, MPOX downregulates the expression of MHC-1 on infected cells and secretes proteins that can target immune molecules such as IL-1*β*, IL8, CCL5, and CCL2. While both Clade 1 and Clade 2 of MPOX can produce proteins that mimic Bcl-2 proteins, which play a crucial role in regulating apoptosis, the Zaire-I-96 strain of MPOX can encode a protein called P1L that can modulate the apoptosis pathway similar to Bcl-2 [68, 70].

### 3.7. Transmission

Mostly by animal-to-human transmission, direct human-to-human transmission through close contact, and indirect human-to-human transmission.

#### 3.7.1. Animal-to-Human Transmission

Until the outbreak of MPOX in 2022, most of the studies were on direct or indirect transmission from infected animals to humans. Besides infecting via direct contact with infected animals, the transmission of the infection through respiratory fluids, fomites, and consumption of infected meats is prominent in the disease [71]. While it is not definitively established that rodents are the main reservoir of MPOX infection, humans are regarded as incidental hosts in the natural environment. In 1964, American giant anteaters (*Myrmecophaga tridactyla*) at the Rotterdam Zoo were infected, most likely due to contact with monkeys [72]. In a 1980 study of 338 MPOX patients (most of them were below 15 years old), over 72% of cases in the DRC were linked to animal interactions [73]. In 2003, unexpected events occurred when exotic rats from Ghana, sold as pets, entered the American pet trade. The virus then spread to prairie dogs, leading to human outbreaks as people came in contact with sick animals and their fluids. This transmission occurred primarily through respiratory droplets and saliva [61]. In several African countries, lifestyles include forest living, hunting, bushmeat handling, and even animal's cage cleaning (noninvasive exposure) and infected animal biting (invasive exposure), which greatly increase the likelihood of animal-to-human transmission of MPOX [34, 74]. These activities increase human–wildlife interaction, therefore increasing exposure to possible animal reservoirs and vectors and helping infectious diseases to spread.

#### 3.7.2. Human-to-Human Transmission

Unlike animal-to-human infection, human-to-human (especially transmitted by respiratory droplets) has a low transmission rate. All 57 healthcare professionals who came into unprotected contact with MPOX in a 2005 cohort study stayed asymptomatic over the observation period [75]. Aside from the possibility of infection spreading through contact with contaminated objects or using the same serving dish as an infected person, direct exposure to respiratory droplets, extended face-to-face interaction, physical contact with affected skin areas, and sexual contact are significant routs of transmitting MPOX between humans [33, 61]. In the 2022 outbreak, the primary mode of human-to-human transmission appears to be through sexual contact. A study in Portugal included 47 male participants, 39 of whom were recognized as MSM. Likewise, six individuals reported engaging in sexual activity with more than ten [76]. In another 2022 observational cohort study that carried out in Bichat–Claude Bernard University Hospital in Paris on 264 patients, 245/295 (95%) of them were men engaged in sexual activities with other men [77].

### 3.8. Clinical Manifestations

After an asymptomatic latent period of approximately 8 days, individuals infected with MPOX exhibit nonspecific prodromal symptoms. These early signs, which precede the characteristic skin lesions, include fever (between 38.5° C and 40.5°C), pruritus, and swollen painful lymph nodes (1–4 cm in diameter) in the submandibular, cervical, and inguinal regions—crucial for differentiating MPOX from smallpox or chickenpox. Typical symptoms within a 95% CI are fatigue, sore throat, difficulty swallowing, headache, backache, cough, photophobia, shortness of breath, nausea, vomiting, and diarrhea [78–82]. Skin lesions, usually 5–10 mm in size, start on the trunk and face and spread centrifugally to peripheral organs such as the palms and soles about 1–3 days after the prodromal phase. They progress from macular rashes to maculopapular, vesicular, and pustular stages and form crusts and scabs within one to two weeks. Lymphadenopathy remains due to the virus's strong immune response [73, 83, 84]. A thorough assessment examining cases before and after the 2022 outbreak found that more than 100 lesions were observed in almost 50% of the patients [79]. Simultaneously with the emergence of skin lesions, lesions also arise in the oral and vaginal regions [85]. It is advised to practice isolation during the initial week of the rash, as the patient is considered contagious until the scabs separate and the throat swab PCR findings show no signs of infection [14]. Immunocompromised and pregnant individuals are more prone to experiencing complications of the condition. These complications include secondary bacterial soft tissue infection, hemorrhagic pustules, pneumonia, and encephalitis [86]. MPOX can be passed from mother to fetus during delivery in pregnant women, potentially resulting in spontaneous miscarriage occurring 14–24 days after the onset of fever. A stillborn fetus may display maculopapular lesions, hepatomegaly, and hydrops fetalis. Postnatal transmission can also happen through close contact while breastfeeding, comparable to the transmission of varicella zoster and Herpes Simplex viruses [87, 88]. However, the clinical manifestations differed slightly during the 2022 outbreak. Several cases exhibited a solitary cutaneous lesion, predominantly localized in the anogenital region (as indicated in the table), and frequently initiated its development in that area. The lesions were frequently characterized by discomfort and did not exhibit the previously reported tendency to spread to other locations. More research is needed to understand the increased sexual transmission of Clade 2 of the virus and its genetic characteristics. Furthermore, in certain instances, cutaneous lesions manifested prior to the commencement of fever [89]. Nevertheless, a significant majority of participants (over 60%) in a cohort study conducted in Spain experienced systemic symptoms before the appearance of a rash [90]. As summarized in Table 1, fever was reported in 53%–72% of patients across different studies, with lymph node swelling being a notable feature in 29%–85% of cases, distinguishing MPOX from other orthopoxvirus infections. Skin lesions displayed a significant regional variation. Genital lesions were the most common, affecting up to 75% of patients in Spain, Portugal, and Canada, as noted by Tarin-Vicente et al. [90] and Sousa et al. [76]. In the United Kingdom and Germany, facial and trunk lesions were more prevalent, with rates between 20% and 55%. These differences highlight the need for tailored diagnostic approaches. Table 1 shows that affected individuals typically have a median age of 35–42 years, with a notable prevalence among MSM in urban areas. Additionally, HIV coinfection rates ranged from 21% to 47%, indicating a need for integrated care for high-risk populations

### 3.9. Complications

MPOX infections can lead to both neurological and psychosocial consequences. According to a Nigerian study conducted in 2020, 25% of patients who were admitted to the hospital exhibited psychiatric symptoms, including anxiety, despair, low mood, and suicidal thoughts [107]. Distinguishing these psychological symptoms from those caused by hospitalization and quarantine presents a considerable challenge. Neurological issues such as seizures, disorientation, and encephalitis have been seen in individuals with MPOX during the 2022 outbreak, with prevalence rates of 2.7%, 2.4%, and 2, respectively [108]. Moreover, brain magnetic resonance imaging (MRI) scans of patients with acute disseminated encephalomyelitis (ADEM) who have received vaccinations reveal normal levels of viruses and detectable specific MPOX IgM antibodies in the analysis of cerebrospinal fluid (CSF) (94–96). During the 2022 outbreak in the United States, two individuals with weakened immune systems developed encephalomyelitis 5–9 days after experiencing initial symptoms [109]. In the cases of systemic MPOX infection, the lack of viral nucleic acid in the CSF, elevated CSF protein levels, and pleocytosis indicate autoimmune responses rather than viral invasion. MRI scans showed brain and spinal lesions in Patients 1 and 2, which suggests MPOX-related proctitis in Patient 2 [109].

MPOX virus causes ocular symptoms in unvaccinated and immunosuppressed people. Eye rashes (14.37%), conjunctivitis (13.89%), redness, discomfort, and discharge (26.9%), conjunctival ulceration (2.13%), lesions (1.62%), and eyelid pustules and pseudopustules (1.08%) are given by pooled prevalence [110].

Severe infections can lead to complications such as myocarditis, epiglottitis, peritonsillar abscess, rectal wall rupture, hemophagocytic lymphohistiocytosis, bronchopneumonia, and necrotizing pneumonia [77, 103, 111]. The immune response to viral fragments impacting the myocardium can cause myocarditis or pericarditis [112]. This phenomenon can also be observed following the administration of the smallpox vaccine [113, 114]. A review study conducted in 2023 on myocarditis caused by MPOX infection found that all cases of myocarditis were accompanied by chest pain and difficulty breathing. Electrocardiographic tests showed ST-elevation in 44% of patients and sinus tachycardia in 22% of patients. Echocardiographic findings indicated a decrease in the ejection fraction in 43% of patients, with an average ejection fraction of 52.14%. Cardiac magnetic resonance (CMR) imaging revealed gadolinium enhancement and edema in 40% of the cases examined [115]. There have been reports of bronchopneumonia and necrotizing pneumonia in humans infected with MPOX. One case involved a 36-year-old man who had HIV, tertiary syphilis, cryptosporidiosis, and Kaposi's sarcoma. Chest radiography showed progressive bilateral nodules, and he developed necrotizing pneumonia as a result of the MPOX infection. Unfortunately, he died from pseudomonas sepsis on the 28th day of the infection [111]. The ICU admission of a 34-year-old Caucasian French woman revealed the presence of acute respiratory distress syndrome, characterized by symptoms such as fever, hypotension, decreased oxygen saturation, and mental abnormalities. The PCR test confirmed that she was infected with MPOX, and the culture of her pleural fluid showed the presence of Streptococcus pyogenes. The high-resolution computed tomography (CT) scans showed significant bilateral consolidation in this case [116].

### 3.10. Treatment

Given the self-limiting nature of MPOX in most cases, conventional antiviral treatment is often unnecessary since many patients can recover with supportive care alone. To reduce the risk of drug resistance and ensure the effectiveness of antiviral drugs, it is essential to limit their use to severe cases or patients at high risk of serious consequences and death, such as those with weakened immune systems due to HIV or chemotherapy with alkylating drugs.

Supportive care management: Behavior, local, and systemic treatments are used to cure MPOX. Avoid contact lenses, use trifluridine or mineral oils on the eyes for pain and lubrication, apply lidocaine gel to moderate skin lesions, and use a cream with amitriptyline 2%, ketamine 5%, and baclofen 5% for painful anogenital lesions. NSAIDs are also given systemically to ease pain [117]. It is advised to cover MPOX cutaneous lesions to stop the disease from spreading directly through them and to gently wash them with soap and water in order to treat MPOX dermal lesions optimally. Patients should also avoid scratching lesions to prevent infection and spread.

Antiviral treatment: The lack of specific antiviral MPOX drugs necessitates using tecovirimat and CDV, which were initially approved for other uses. Understanding the virus's structure and critical proteins is crucial for developing targeted antiviral therapies.

Tecovirimat (ST-246), also known as TPOXX®, is an antiviral medication specifically developed to inhibit the VP37 protein, which is produced by the F13L gene. This gene is responsible for conferring resistance to BCV in orthopoxviruses, including vaccinia and MPOX. This protein is crucial for creating extracellular enveloped virions (EEVs) by assisting in encasing intracellular MVs (IMVs) with the lipid membrane of the host cell. Tecovirimat inhibits the development of MVs that can leave the cell and disseminate the virus by binding to VP37 and blocking its interactions with GTPase, Rab9, and TIP47, thus restricting the infection [118]. This antiviral medication was authorized by the FDA in 2018. It is offered in two dosage forms: a 600-mg oral capsule taken twice a day and a 200-mg intravenous infusion also taken twice a day. This treatment is the principal treatment for severe instances of MPOX and individuals at high risk of severe disease, such as those with a weakened immune system and asplenia, or who are receiving alkylating drugs. Significantly, the oral formulation demonstrates increased absorption when consumed with food in a fed condition [119]. However, the efficacy of this drug can be compromised by resistance due to a mutation at Amino Acid Position 277 [120]. In a 2023 report, 84.6% of patients receiving tecovirimat intravenously experienced at least one adverse effect. The most common were pain at the infusion site (73.1%), swelling (38.5%), and erythema (23.1%). Headaches and dizziness were also reported by 26.9% and 23% of patients, respectively. Other side effects such as myalgia, back pain, diarrhea, and photophobia were rare, occurring in less than 5% of cases [121]. No renal dose adjustment needed in the capsule formulation of tecovirimat, while its IV formulation is contraindicated in CrCl below 30 mL/min due to B-cyclodextrin existence in formulation [122]. Prairie dogs, macaque monkeys, and cynomolgus monkeys (*Macaca fascicularis*) are the examples of tecovirimat's therapeutic applications [123–126]. In a 2011 study, Prairie dogs (*Cynomys ludovicianus*) infected with the Congo basin clade were administered intranasally with tecovirimat, and it was celebrated that 100% of cases improved and became asymptomatic without any instances of death [125]. Before that, in a 2009 study, intravenous administration of tecovirimat at a dose of 3 mg/kg/day, three days postinfection, established a zero percent mortality rate in cynomolgus monkeys infected with the West African clade [126]. It is also worth mentioning that there are no reports of fetal toxicity regarding this medicine [122].

CDV (VISTIDE®), also known by its IUPAC designation [(2S)-1-(4-amino-2-oxopyrimidin-1-yl)-3-hydroxypropan-2-yl]oxymethylphosphonic acid, is a pharmaceutical compound that acts as a cytosine nucleotide analog and inhibits the activity of DNA polymerase. It has been granted approval by the FDA for the treatment of cytomegalovirus (CMV) retinitis in individuals who are infected with HIV [127]. Upon cellular uptake, CDV undergoes phosphorylation by the host cell's enzyme, resulting in the formation of CDV diphosphate (CDV-pp), which is its active form. This active form affects the elongation of the DNA chain during replication by competitively limiting the inclusion of deoxycytidine triphosphate [128]. Due to inadequate absorption in the gastrointestinal (GI) tract, pharmacists were compelled to produce an intravenous dosage form with a recommended dose of 5 mg/kg once weekly for the treatment of MPOX infection [129]. The most significant adverse effect of CDV is nephrotoxicity, which is dose-dependent. This is characterized by the presence of proteinuria followed by glucosuria, an increase in the blood creatinine level, and a decrease in the serum bicarbonate concentration [122, 130]. Additional documented adverse effects comprise neutropenia, ocular hypotony, iritis, uveitis, and pyrexia [130]. Misuse of different antimicrobial medications may lead to the development of drug-resistant strains in the same group of microorganisms. The susceptibility of the MPOX virus to resistance is a significant trouble. The emergence of CDV-resistant species among orthopoxviruses, including MPOX, complicates the situation further [131]. The rise of CDV-resistant orthopoxviruses, such as smallpox, vaccinia, and MPOX, presents a major health risk due to specific mutations found in the *E9L* (DNA polymerase) and *F13L* (viral membrane protein) genes of vaccinia virus. Similar genetic variations, specifically *A314V* and *A684T*, have also been observed in MPOX virus. These genetic changes result in reduced ability to cause disease and replicate efficiently, requiring ongoing monitoring of the genome, the creation of different treatment options, and additional investigation into the specific molecular processes and adaptability of resistant strains [132, 133].

In 2021, the FDA-approved BCV (marketed as Tembexa®) for treating smallpox. This medication enhances the lipophilicity and cellular permeability of CDV by including a hexadecyloxypropyl group [134]. This anti-dsDNA virus drug hydrolyzes to CDV after entering into host's cells and is then phosphorylated to CVD-pp to the inhibition of DNA replication. The coadministration of tecovirimat with BCV can reduce the effective concentration (EC_50_) of the latter [135]. BCV has a long half-life similar to CDV but with reduced nephrotoxicity due to its lipophilicity. Additionally, BCV has significant concentrations in the lungs, spleen, and liver [136–138]. BCV has the potential to raise liver enzyme levels, particularly alanine aminotransferase (ALT), and raise bilirubin levels, which can cause damage to liver cells (hepatocellular toxicity) [139, 140]. The fasting state reduces BCV's orally absorption and plasma concentration [141]. BCV is not a recommended drug for pregnant or breastfeeding women.  Table 2 compares therapeutic agents for MPOX, including their mechanisms of action, pharmacokinetics, adverse effects, and safety profiles. Tecovirimat is versatile and safe for various populations, while BCV requires hepatic monitoring, and CDV demands renal precautions. This analysis aids clinicians in tailoring treatments based on patient-specific needs and contraindications.

Immunoglobulin therapy involves the administration of antibodies to enhance the immune response against specific pathogens. The CDC recommends Vaccinia Immune Globulin Intravenous (VIGIV) for the treatment of orthopoxviruses infections, and it has received FDA approval in 2005 for managing complications associated with vaccinia vaccination [142]. Derived from the plasma of healthy donors, this treatment involves aggregated polyclonal immunoglobulin and has shown cross-neutralization activity against the MPOX virus in a Macaca mulatta model infected with the CB clade of MPOX, effectively inhibiting the virus from infecting additional cells, as depicted in Figure 2 [40, 143].

**Figure 3:**
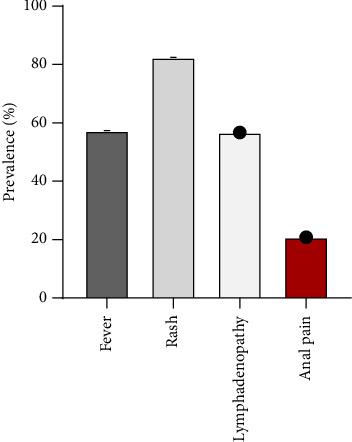
Prevalence of key clinical manifestations in MPOX patients during the 2022 outbreak. Based on Figure 1, fever (57.37%), rash (88.44%), lymphadenopathy (56.68%), and anal pain (20.81%) were among the most frequently reported symptoms during the 2022 MPOX outbreak, highlighting variations in the severity and occurrence of these clinical manifestations.

Imatinib, a drug primarily used for chronic myeloid leukemia, has shown potential as an antiviral agent by disrupting actin dynamics and preventing viral egress. This could reduce the spread of the virus by inhibiting the release of EEVs (see Figure 3). Other medications have shown effectiveness against MPOX besides imatinib. In a 2017 experiment, resveratrol, found in plants such as grapes, peanuts, and blueberries, hindered virus reproduction by disrupting DNA synthesis [144]. Ribavirin, a medication commonly used to treat hepatitis C (HCV), has also shown effectiveness against MPOX and other orthopoxviruses infections [128, 144]. In infected cells treated with recombinant INF-beta (INF-*β*), the antiviral protein myxovirus resistance protein A (MxA) was induced, and it was exhibited that the continuous expression of MxA inhibits MPOX infection [145]. Further investigations have been carried out to find out the antiviral properties of recombinant beta INF against the MPOX virus.

For numerous infectious diseases, including MPOX infection, new medication formulations and techniques of administration are in development. A striking example comes from a 2024 study on a poly(lactic-co-glycolic acid) (PLGA) core-enclosed TPE-BT-DPTQ. We further refined the core, combined it, and extruded it under a macrophage membrane. We recovered the membrane by means of mechanical fragmentation, ultracentrifugation, and hypotonic lysis among other techniques. The result of this technique was the final biomimetic nanoparticles (BNPs), sometimes referred to as TBD@M NPs. In this work, photothermal BNPs administered to mice infected with MPOX produced a notable reduction in viral titers within the lesions, accelerated healing of the lesions, and effective inhibition of viral transmission [146].

### 3.11. Vaccination

Because orthopoxviruses share genetic similarities, the immune response to one can help protect against others. Smallpox vaccines can prevent MPOX [147–149]. There are three generations of vaccines against smallpox. The most significant of the first generation of these vaccines, which successfully prevented person-to-person transmission in Africa, is Dryvax® [150]. The production of Dryvax® was discontinued in the United States in 1978 after 15 million doses had been produced [151, 152]. Other first-generation vaccines utilizing a live-unattenuated platform, which are no longer licensed, include Lister-based vaccines and the New York City Board of Health (NYCBH) [152].

ACAM2000®, a second-generation vaccine developed by Emergent BioSolutions, is a live-attenuated, replication-competent vaccinia virus in lyophilized form. After receiving FDA approval in 2007 [61, 153], this vaccine was introduced as an alternative to Dryvax® and approved for 18–64-year-old individuals, exhibiting similar safety profiles and complications, including progressive vaccinia, eczema vaccinatum, and encephalitis [33, 154]. ACAM2000®, administered for postexposure prophylaxis with a bifurcated needle, provides peak protection 28 days after vaccination, with reimmunization advised every three years for high-risk exposures and every 10 years for less virulent strains like cowpox [155]. Immunosuppressed populations, such as those suffering from leukemia, lymphoma, HIV infection, patients with metastatic malignancies, transplant recipients, and undergoing cancer chemotherapy and radiotherapy individuals or taking corticosteroids with a dose of ≥ 2 mg/kg for ≥ 2 weeks and TNF inhibitors (e.g., adalimumab and infliximab), are in a group for whom this vaccine is not recommended [155]. Having cardiac risk factors such as diabetes, smoking, hypertension, or hyperlipidemia is a contraindication for ACAM2000® due to the high risk of myopericarditis, which can present with symptoms such as dyspnea, chest pain, palpitations, and ECG abnormalities in these individuals [155, 156].

Unlike ACAM2000®, which is derived from a clonal isolate of Dryvax [33], LC16m8 is a third-generation vaccine derived from the first-generation Lister strain incorporating a deletion in the B5R immunogenic membrane protein [157], produced in cell culture using rabbit kidney cells [158]. This minimally replicating vaccinia virus vaccine has demonstrated effectiveness in postexposure prophylaxis when administered within 4–14 days after close contact, regardless of prior smallpox vaccination, and it is the only vaccine authorized for use in children among the three MPOX vaccines [159].

Another third-generation vaccine, Modified Vaccinia Ankara (MVA), branded as IMVANEX® (in Canada) and JYNNEOS® (in the United States), has demonstrated adequate cellular and humoral immunity in animal models [160]. The vaccine is administered intradermally in two doses, specifically on Day 0 and Day 28, by FDA guidelines. This vaccine is exceptionally safe for individuals with compromised immune systems, as it does not use live viruses, significantly reducing the risk of infection [161].

In recent years, mRNA-based vaccines have been developed and introduced as novel vaccines, with high efficacy in triggering both humoral and cellular immune responses [162] and with faster production against tumor cells and viruses [155]. Self-amplifying RNA (saRNA) [163] and nonreplicating mRNA vaccines [164] are two types of RNA vaccines. saRNA vaccines contain the code for both the target antigen and a replicase complex [165], while nonreplicating vaccines contain the code for only the target antigen [155]. In addition to viral infections [166], the applications of saRNA vaccines have expanded to include bacterial infections [167], parasitic infections [168], and cancers (through passive vaccination with monoclonal antibodies encoding) [169, 170]. The MPOX is a new target for this novel vaccine technology. For instance, in Sang and colleagues' study, the immunogenicity of an MPOX virus quadrivalent mRNA vaccine, which was prepared using A29L, A35R, M1R, and B6R as antigen targets, was evaluated in mice. As a result, they observed a strong cellular and memory cell immune response against MPOX without any significant rise in inflammatory cytokines or histopathological changes in the mice [171]. A multiepitope-based vaccine is another novel approach against MPOX, which Shantier and coworkers have developed. This safe, innovative vaccine elicits an immune response against the MPOX by utilizing cell surface binding proteins, without causing side effects [172].

### 3.12. AI and MPOX

Deep learning (DL) is a specific branch of machine learning (ML) that falls within the broader field of AI. It is distinguished by its capacity to utilize neural networks with numerous layers to identify and represent intricate patterns in large datasets. DL has gained significant recognition for its revolutionary applications in the field of medical science. Significantly, it aids in the identification of illnesses by allowing the examination of various imaging techniques, spanning from basic skin pictures [173] to advanced diagnostic instruments such as CT scans [174] and MRIs [175], largely utilizing convolutional neural networks (CNNs). Furthermore, DL plays a crucial role in the detection of pathogens via genomic sequencing. Utilizing recurrent neural networks (RNNs) improve diagnostic accuracy. Recent research on highly transmissible infectious diseases like MPOX has focused on DL to examine skin lesions and detect them early. In a 2022 study, MobileNetv2 showed the highest accuracy at 91.11% in binary classification with a sample size of 169. VGG16 and VGG19 are CNN models trained on ImageNet, while ResNet50 and ResNet101 are residual networks [176]. The Vision Transformer (ViT), introduced by Dosovitskiy et al., is a pretrained model for image classification that treats images as sequences of patches processed by a transformer encoder (TE), with a multilayer perceptron (MLP) normalizer, multihead attention mechanism (MHAM), and embedded patches (EPs) forming its architecture [177]. This new model has uses outside the ones described. In a study by Chaturvedi et al. on the epidemiological and behavioral elements influencing MPOX transmission, it was proposed that, much as with smallpox vaccination, variations in transmission probability—whether sexual or nonsexual—had a significant impact on MPOX in their model. Furthermore, in the absence of pre-exposure vaccination, contact tracing's precision and timeliness proved to be quite important in reducing MPOX flow within the model [178]. Drug discovery and vaccine production through molecular modeling and virtual screening, personalized treatment aimed at achieving the most therapeutic effect with fewer adverse events, and optimal public health policies are just a few examples of how AI can play a role in medical science.

### 3.13. Policy Implications and Future Research Studies

Policymakers should focus on vaccination campaigns targeting high-risk groups, including healthcare workers, MSM, and immunocompromised individuals, particularly in regions with Clade 1b cases. Establishing genomic surveillance systems is also essential for monitoring MPOX strain evolution and enabling proactive health responses.

Despite progress, gaps remain in MPOX research. The long-term efficacy of existing smallpox vaccines against MPOX is still unclear, and AI diagnostic tools need real-world validation. The transmission dynamics among MSM highlight the importance of understanding demographic factors for effective public health interventions. High rates of HIV coinfection in MPOX patients indicate the need for integrated care approaches.

Future research should prioritize the development of next-generation MPOX-specific vaccines, mainly using mRNA technologies, and expand the use of AI for outbreak surveillance. Addressing transmission's social and behavioral aspects is crucial for reducing stigma and promoting preventive measures. Evaluating current public health policies will provide valuable insights for refining strategies for future outbreaks. By addressing these issues and utilizing innovative tools, global health systems can enhance their preparedness and response to MPOX and other emerging infectious diseases.

## 4. Conclusion

The COVID-19 pandemic and the 2022 MPOX outbreak highlighted the urgent need to handle viruses and their potential to cause widespread epidemics and devastation [1]. Developing specific treatments and effective vaccinations for viruses such as SARS-CoV-2, MPOX, Zika virus, and Ebola can be challenging due to mutations that alter transmission patterns and clinical properties. This review contributes to this field by providing comprehensive data on various aspects of MPOX infection.

Modern medications can be developed with a thorough knowledge of viral structures and the protein and nonprotein components engaged in their pathogenesis and life cycle This knowledge enables the design of drugs against them. Many of these structures can be addressed by medications used for different purposes; network pharmacology helps one to find these targets [179]. Monoclonal antibodies and new chemical therapies can target these structures. Raising public awareness about immunization, preventive measures, and sexual health behaviors is essential, especially for gay individuals and those with multiple sexual partners.

Combining traditional knowledge of diseases with modern AI technology can help us tackle the challenges of MPOX more effectively. Policymakers, doctors, and researchers must work together to create strategies that improve preparedness, lower the risks of outbreaks, and enhance patient care. The global health community can strengthen its response to MPOX and other emerging infectious diseases by finding and addressing gaps in our current approach [180].

## Figures and Tables

**Table 1:** Clinical manifestation during 2022 outbreak.

Authors, year, reference	Country	Study type	No. of patients	Age median	HIV positive (∼%)	Skin rash (∼%)	Site of skin lesion (∼%)	Fever (∼%)	Lymph node swelling (∼%)	Proctitis or anal pain (∼%)
Angelo et al. 2022 [91]	Multicountry	Cross-sectional	226	37	92 (41)	137 (61)	Genital (46), perianal (28), trunk (27), face (23), mouse or oral mucosa (20), palms and hands (11)	131 (58)	82 (36)	33 (15)
Català et al. 2022 [92]	Spain	Prospective cross-sectional	185	38.7	78 (42)	185 (100)	Genital (53) face (39) perianal (34) thorax (25), plantar (12), palmar (6)	100 (54)	104 (56)	40 (22)
Gomez-Garberi et al. 2022 [93]	USA^a^	Prospective observational	14	42	8/13 (62)	14 (100)	Anogenital (100), face (50)	6 (43)	8 (57)	1 (7)
Girometti et al. 2022 [94]	UK^b^	Retrospective observational analysis	54	41	13 (24)	54 (100)	Genital (61), perianal (44), face (20), limbs (50)	31 (57)	30 (56)	NR^c^
Harrison et al. 2023 [95]	Canada	Cohort	346	37	NR	314 (91)	Genital (46.9), anal (36.7), palmar (26.9), face (34), plantar (16)	166 (52)	204 (64)	102 (33)
Hennessee 2022 [96]	USA	Case series	83	11.5	2 (2)	83 (100)	Anogenital (40), trunk (55), face and head (41), limbs (31)	29 (35)	24 (29)	NR
Hornuss et al. 2022 [97]	Germany	Case series	4	37.7	0 (0%)	3 (75)	Anogenital (100), face (50)	2 (50)	2 (50)	NR
Inigo Martinze et al. 2022 [98]	Spain	Cohort	508	35	225 (44)	498 (98)	Anogenital (72.1), face (35.5), palms or plants (24.9)	324 (64)	311 (61)	81 (16)
Moschese et al. 2022 [99]	Italy	Case series	4	33	2 (50)	3 (75)	Anogenital (50), trunk (100), face (100)	4 (100)	4 (100)	1 (25)
Nörz et al. 2022 [100]	Germany	Case series	10	33	2 (20)	10 (100)	Anogenital (90), trunk (40), face (20)	3 (30)	5 (50)	NR
Patal et al. 2022 [101]	UK	Retrospective observational	197	38	70 (35)	197 (100)	Genital (56.4), anus or perianal (41.6), face (36), trunk (35.5), hands or feet (28.4)	122 (62)	114 (58)	71 (36)
Philpott et al. 2022 [102]	USA	Cohort	1195	35	136/334 (41)	1004 (100)	Genital (46.4), arms (39.6) perianal (31.3) trunk (21.7) face (38.4), soles or feet (10.7)	596 (63)	545 (58)	NR
de Sousa et al. 2022 [76]	Portugal	Retrospective observational	47	35.1	21 (45)	47 (100)	Genital (63.8), anorectal (46.8), trunk (44.7), face (27.7)	25 (53)	32 (68)	NR
Tarin-Vicente et al. 2022 [90]	Spain	Multicenter prospective observational	181	37	72 (40)	181 (100)	Genital (55) perianal (36) trunk (57), hand and feet (60)	131 (72)	153 (85)	45 (25)
Thornhill et al. 2022 [103]	Multicountry	Case series	528	38	218 (41)	500 (95)	Anogenital (75), face (25), trunk or limbs (55), palms or soles (10)	330 (62)	295 (56)	75 (14)
Hoffman et al. 2023 [104]	Germany	Multicenter retrospective	546	39	256 (47)	NR	Genital (49.9), anal (47.9), trunk or extremities (37.5)	272 (53)	213 (43)	NR
Mailhe et al. 2023 [77]	France	Cohort	264	35	73 (29)	258 (98)	Genital (54), perianal (40), face (35), limbs (48)	171 (68)	174 (69)	45 (18)
van Ewijk et al. 2023 [105]	The Netherlands	Epidemiological surveillance	1000	37	187 (21)	914 (92)	Genital (51) perianal (33) face (34) trunk (38), limbs (51)	521 (53)	371 (37)	178 (18)
Hoffman, Jessen, and Boesecke, 2022 [106]	Germany	Retrospective	301	39	141 (47)	276/301 (99)	Genital (49), anal (51), oral, perioral, head (24), trunk (42)	168/274 (61)	116/263 (44)	NR

*Note:* Clinical manifestations of MPOX during the 2022 outbreak across different studies. This table summarizes the prevalence of clinical manifestations, including proctitis, fever, lymph node swelling, and skin lesions, as reported in various studies during the 2022 MPOX outbreak.

^a^United States of America.

^b^United Kingdom.

^c^Not reported.

**Table 2:** Comparison of characteristics of different therapeutic agents for MPOX infection.

Characteristic	Tecovirimat	Brincidofovir	Cidofovir
Mechanism of action	Inhibits the viral envelope protein VP37, preventing the release of mature virus particles from infected cells, thus limiting viral spread	DNA polymerase inhibitor	DNA polymerase inhibitor
*t*_(1/2)_	18–26 h	19.3 h (CDV-pp: 133 h)	3.2–4.4 h
Protein binding	77%–82%	> 99.9%	< 6%
Elimination	Less than 1% exsert in urine as unchanged drug; fecal elimination	More than 50% excreted in urine and 40% in feces after metabolism	70%–85% excreted in urine unchanged
Significant adverse drug effect	Headache, abdominal pain, nausea, vomiting, oral dryness, hypersensitivity	Nausea, diarrhea, vomiting, abdominal pain, and bilirubin and transaminase elevation	Neutropenia, nephrotoxicity, decrease in ocular pressure
Pharmacokinetic properties and administration	Taking 30 min after a moderate-to-high fat meal increases absorption. For children 13 kg and above, the capsule can be opened and mixed with milk or soft food.	The tablet should be taken on an empty stomach or with a low-fat meal. Shake the suspension form before taking. It can be given through an NG/G tube	Dilute in 100 mL NS and infuse over 1 h WITH probenecid: 2 g 3 h before CVD, 1 g at 2- and 8 h postcompletion. Administer 1L NS with each CVD infusion over 1-2 h before infusion. Consider additional NS infusion at the start or after CVD over 1-3 h if tolerable
Renal dose adjustment	No dose adjustment is needed for capsules; IV form is contraindication in CrCl < 30 mL/minute	Non	Reduce maintenance dose from 5 mg/kg to 3 mg/kg if SCr increases 0.3–0.4 mg/dL from baseline and discontinue if ≥ 0.5 mg/dL above baseline or development of ≥ 3+ proteinuria
Use in pregnancy	No fetal or embryo toxicity observed in animal studies	Fetal harm and embryo toxicity were observed in rats and rabbits. Pregnancy tests should be done before administration, and contraceptives should be used for 2 months after the last dose	Not recommended in pregnancy
Use in lactation	Presenting in milk is unknown; tecovirimat is preferred antiviral in lactating patients	It is not recommended as an alternative therapy in MPOX lactating patient	Not recommended in lactation
Metabolism	CYP2C19, CYP2C8 weak inhibitor; CYP3A4 weak inducer	MRP2 inhibitor	MRP2 inhibitor
Safety profile	Mild side effects (headache, nausea). Safe for pregnancy/lactation. Avoid IV use in severe renal impairment. Preferred for immunocompromised patients	Risk of hepatotoxicity (elevated liver enzymes). Contraindicated in pregnancy/lactation. Requires liver function monitoring. Avoid severe liver dysfunction	Significant nephrotoxicity. Requires hydration and probenecid coadministration. Contraindicated in severe renal impairment, pregnancy, and lactation. Close renal monitoring essential

*Note:* Comparative characteristics and safety profiles of therapeutic agents for MPOX. This table provides a side-by-side comparison of key therapeutic agents for MPOX, including their mechanisms of action, half-lives, significant adverse effects, and safety profiles for specific populations.

## Data Availability

The data that support the findings of this study are available from the corresponding author upon reasonable request.
